# Comparison between UK Biobank and Shanghai Changfeng suggests distinct hip morphology may contribute to ethnic differences in the prevalence of hip osteoarthritis

**DOI:** 10.1016/j.joca.2023.10.006

**Published:** 2023-11-05

**Authors:** Jiayi Zheng, Monika Frysz, Benjamin G. Faber, Huandong Lin, Raja Ebsim, Jieyu Ge, Yanling Yong, Fiona R. Saunders, Jennifer S. Gregory, Richard M. Aspden, Nicholas C. Harvey, Bing-Hua Jiang, Timothy Cootes, Claudia Lindner, Xin Gao, Sijia Wang, Jonathan H. Tobias

**Affiliations:** # CAS Key Laboratory of Computational Biology, Shanghai Institute of Nutrition and Health, University of Chinese Academy of Sciences, Chinese Academy of Sciences, Shanghai, China; † Musculoskeletal Research Unit, University of Bristol, Bristol, UK; ‡ Medical Research Council Integrative Epidemiology Unit, University of Bristol, Bristol, UK; § Department of Endocrinology and Metabolism, Zhongshan Hospital, Fudan University, Shanghai, China; ¶ Fudan Institute for Metabolic Diseases, Shanghai, China; ∥ Division of Informatics, Imaging and Data Sciences, The University of Manchester, Manchester, UK; ## Academy of Medical Science, Zhengzhou University, Zhengzhou, China; †† Centre for Arthritis and Musculoskeletal Health, University of Aberdeen, Aberdeen, UK; ‡‡ Medical Research Council Lifecourse Epidemiology Centre, University of Southampton, Southampton, UK; §§ NIHR Southampton Biomedical Research Centre, University of Southampton and University Hospital Southampton NHS Foundation Trust, Southampton, UK

**Keywords:** Hip morphology, DXA, Radiographic hip osteoarthritis, Ethnic differences

## Abstract

**Objects:**

Joint morphology is a risk factor for hip osteoarthritis (HOA) and could explain ethnic differences in HOA prevalence. Therefore, we aimed to compare the prevalence of radiographic HOA (rHOA) and hip morphology between the predominantly White UK Biobank (UKB) and exclusively Chinese Shanghai Changfeng (SC) cohorts.

**Methods:**

Left hip iDXA scans were used to quantify rHOA, from a combination of osteophytes (grade ≥1) and joint space narrowing (grade ≥1), and hip morphology. Using an 85-point Statistical Shape Model (SSM) we evaluated cam (alpha angle ≥60°) and pincer (lateral centre-edge angle (LCEA) ≥45°) morphology and acetabular dysplasia (LCEA < 25°). Diameter of femoral head (DFH), femoral neck width (FNW), and hip axis length (HAL) were also obtained from these points. Results were adjusted for differences in age, height, and weight and stratified by sex.

**Results:**

Complete data were available for 5924 SC and 39,020 White UKB participants with mean ages of 63.4 and 63.7 years old. rHOA prevalence was considerably lower in female (2.2% versus 13.1%) and male (12.0% and 25.1%) SC compared to UKB participants. Cam morphology, rarely seen in females, was less common in SC compared with UKB males (6.3% versus 16.5%). Composite SSM modes, scaled to the same overall size, revealed SC participants to have a wider femoral head compared to UKB participants. FNW and HAL were smaller in SC compared to UKB, whereas DFH/FNW ratio was higher in SC.

**Conclusions:**

rHOA prevalence is lower in Chinese compared with White individuals. Several differences in hip shape were observed, including frequency of cam morphology, FNW, and DFH/FNW ratio. These characteristics have previously been identified as risk factors for HOA and may contribute to observed ethnic differences in HOA prevalence.

## Introduction

Hip osteoarthritis (HOA) is a significant cause of pain, disability, and societal cost in older individuals. Prior research has reported a lower HOA prevalence in older Chinese individuals compared to Caucasians.^1,2^ Recent studies have also reported a relatively low prevalence of radiographic HOA (rHOA) in other Asian countries.^3–6^ Such ethnic differences in HOA prevalence are likely to arise from a combination of environmental and genetic factors, though the precise mechanisms involved remain unclear.

Hip shape, quantified using a variety of X-ray-based methods, is an important risk factor for HOA.^7^ One of the most studied changes is cam and pincer morphology, both of which are thought to contribute to femoroacetabular impingement. For example, cam morphology, a bulge overlying femoral head-neck junction, was related to the risk of HOA in a previous longitudinal study.^8^ Moreover, a prospective cohort study found that cam morphology played a significant role in early osteoarthritis (OA) and could contribute to HOA in later life.^9^ A recent cross-sectional study in UK Biobank (UKB) found that cam morphology, assessed on hip dual-energy X-ray absorptiometry (DXA) scans, was associated with osteophytosis throughout the hip.^10^ However a subsequent Mendelian Randomisation study suggested that cam morphology may be a feature of HOA in older adults, rather than having any causal role.^11^ In contrast, we recently found pincer morphology, causing over-coverage of the femoral head by the acetabulum, to have no association with rHOA or hip pain.^10^ Acetabular dysplasia, defined as under-coverage of the femoral head, was reported as a risk factor in a few prospective studies with limited sample sizes.^8^

Hip geometry, derived from the size and relative proportions of different components of the hip, may convey additional information with respect to HOA risk. For example, femoral neck width (FNW) was found to be related to an increased risk of HOA,^12^ suggesting that bone size-related parameters and OA might share a common cause, such as genes regulating bone growth. In our recent DXA-based study in UKB, femoral head width relative to FNW was also found to predict the risk of HOA.^13^

Little is known about ethnic differences in hip shape. However, if significant differences exist, it is possible that these contribute to those in HOA prevalence. An improved understanding of how hip shape differences contribute to HOA may help to identify adverse biomechanical influences that lead to HOA, which are potentially amenable to physiotherapy, orthotics, or even surgery.^7^ It may also be possible to harness a greater understanding of these relationships in developing prediction rHOA tools.^14^ Therefore, to gain a better understanding of the relationship between hip shape and HOA in different populations, in the present study, we aimed to ascertain the prevalence of rHOA in Chinese adults from the Shanghai Changfeng (SC) cohort and compare this with that of Caucasians and other ethnic groups in the UKB study. Subsequently, we aimed to compare hip shape and morphology between these cohorts and infer to what extent any differences that were observed might contribute to those in rHOA prevalence.

## Participants and methods

### Study population

SC is a prospective community-based cohort study, in the Changfeng community of the Putuo District in Shanghai, China (N = 6595, aged ≥45 years) between June 2009 and December 2012.^15^ SC is organised and directed by the Fudan-Erasmus Research Institute of Medicine, which is a joint venture between Zhong Shan Hospital of Fudan University in Shanghai and the Erasmus Medical Center, Rotterdam, the Netherlands. Ethics approval was granted by the ethics committee of Zhongshan Hospital affiliated to Fudan University (B2008-119(3)) and written informed consent was provided by all participants before participation. All participants answered a survey and were invited for a DXA scan of their left hip using a high-resolution iDXA (GE Lunar), the majority of whom attended (N = 6082).

UKB is a prospective nationwide multi-centre cohort study of half a million participants, most of whom are of European ancestry. Participants self-identified on the questionnaire as different ethnicities which were grouped into the following categories: White, Black, Asian (including Indian, Pakistani, and Bangladeshi), mixed-race, Chinese, and other. Only those identified as White, Black, Asian, or Chinese were included in this study. Hip iDXA scans (GE-Lunar, Madison, WI) were collected as part of the UKB imaging enhancement study which commenced in 2014.^16^ The UKB Ethics Advisory Committee oversees the maintenance, development, and use of UKB data and its approval covers this study (application number 17925). UKB received ethics approval from the National Information Governance Board for Health and Social Care and North West Multi-centre Research Ethics Committee (11/NW/0382). All subjects provided informed consent before participation. Participants are being invited for a DXA scan several years after inception, with the expectation that 20% will be scanned in total.

### Statistical hip shape model

In brief, the left hip, excluding osteophytes, was outlined in both UKB and SC DXAs using 85 points placed by a machine-learning trained software (BoneFinder^®^, The University of Manchester). Point annotations were reviewed and corrected where necessary. Following point placement, hip shape size and rotation were standardised by Procrustes analysis. Principal components analysis was then used to build a statistical shape model (SSM) from all available images in UKB, producing a set of orthogonal modes of variation. Further analysis focused on the first ten hip shape modes (HSMs), which explain 86.3% of hip shape variance (see Supplementary Fig. 1). Using the SSM built in UKB and the existing shape modes, all available images in SC were analysed to get comparable mode scores. A more detailed description of the methods, including positions of the 85 points outlining the hip, is provided in our previous publication.^17^

### Radiographic hip osteoarthritis (rHOA)

A DXA-based atlas (the University of Bristol) was used to annotate osteophytes at the lateral acetabulum, superolateral femoral head, and inferomedial femoral head. Two trained annotators (JZ, PhD student, YY, Masters student) checked all SC DXAs and marked all osteophytes together using methods previously described.^18^ If there was disagreement then a third opinion was gained from an experienced annotator (BGF, Rheumatologist). A random subsample of images (n = 100) was assessed individually by all three annotators which showed an inter-rater Kappa of 0.7 with an agreement of 90% for the presence of osteophytes. Osteophyte semi-quantitative grades (0–3) were automatically calculated based on osteophyte area thresholds (grade 1: ≥1 mm^2^; grade 2: ≥10–19 mm^2^ depending on location); grade 3 osteophytes ≥50 mm^2^. Minimum joint space width (mJSW) was calculated using a custom Python script^19^ between the acetabulum (points 78–84) and superior femoral head (points 22–31) as follows: A segment is created by drawing a straight line between two neighbouring points, for example, two points on the acetabulum. Then the shortest distance is calculated between this line and an opposing point, in this example on the femoral head. The automated method repeats this process for all segments and points selected, and the shortest distance representing mJSW (in mm) is saved.^10^ Subsequently, joint space narrowing (JSN) was semi-quantitatively graded (0–3) based on height-adjusted mJSW thresholds. rHOA grades were defined using a score combining osteophyte and JSN grades.^20^ Overall rHOA grade (0–4) was generated using cut-offs, from the sum of osteophyte grades (0–3) at the three locations and JSN grades (0–3), as follows: rHOA grade 0 (sum = 0), grade 1 (sum = 1), grade 2 (sum = 2–3), grade 3 (sum = 4–6), grade 4 (sum = 7–12).

### Cam/pincer morphology and acetabular dysplasia

Previously validated automated methods were used to automatically measure alpha angle (AA) and lateral centre-edge angle (LCEA) using the outline points.^10,19,21^ Cam morphology was defined as AA ≥60°, and pincer morphology was defined as LCEA ≥45°. Acetabular dysplasia was defined as LCEA < 25°.

### Hip geometry

Custom Python 3.0 scripts were developed and used to automatically derive FNW, hip axis length (HAL), and diameter of femoral head (DFH), as previously described.^13^ In brief, FNW was defined as the shortest distance measured between the superior and inferior side of the femoral neck, with a line-segment approach used to automatically calculate the narrowest distance between the relevant points. DFH was defined as the distance across the spherical aspect of the femoral head. To estimate this, a circle of best fit was placed around the femoral head, with the diameter of the circle taken to represent the DFH in mm. HAL was defined as the distance from the base of the greater trochanter to the medial aspect of the femoral head, drawn through the centre of the circle of best fit (used to calculate DFH), in millimetres. All images with values lying beyond ± 2 standard deviations (SDs) from the mean were reviewed manually.

### Statistical methods

The distributions of age, height and weight for each ethnic group are shown as means and SDs. One-way Analysis of Variance (ANOVA) was used when comparing age, height, and weight between SC and UKB subgroups. The distribution of continuous hip parameters, both distance and angle phenotypes, are also shown as means and SDs, including mJSW, AA, LCEA, FNW, HAL, and DFH. Again, we used one-way ANOVA to compare data distributions between SC and UKB subgroups. Whether the observations were in Gaussian distribution was checked by Shapiro-Wilk test. If some phenotypes did not follow Gaussian distributions, the Kruskal-Wallis test by ranks was used. If some comparisons had unequal variances examined by the homogeneity of variance test, Welch’s ANOVA was used. Chi-squared test was used to examine any differences in rHOA, JSN, and osteophyte categories between SC and UKB subgroups. Fisher’s exact test was used when over 20% of the rHOA, JSN and osteophyte grade had a sample size of less than 5. Results were stratified by sex, given previously observed sex differences in proximal femur shape.^22^ Bonferroni correction was performed to adjust for multiple comparisons. To compare HSMs and hip geometric measures accounting for the differences in age, height, and weight between ethnic groups, these were both centred on age, height, and weight of White UKB participants (in contrast to geometric measures, HSMs are scaled to the same size by Procrustes analysis, however, this may not fully account for effects of height and weight on the hip shape). To do this we used ethnic-specific beta coefficients derived from multiple linear regression analyses of age, height, and weight on HSMs and geometric parameters in SC and UKB. Data analysis was performed in SPSS Statistics 24.

## Results

### Characteristics of participants

After excluding those with low-quality images (n = 62), incomplete scans (n = 74), and femurs with a previous history of fracture or total hip replacement (n = 8), the study included 5924 participants (3417 women, 2507 men) from SC. Similarly, after removing 820 images due to either poor image quality, image error, or withdrawal of consent, the study included 39,826 participants (20,744 women, 19,082 men) from UKB. All participants were over 45 years old, with an average age of 63.4 years old in SC and 63.7 years old in UKB. SC participants were similar in age to White UKB participants, but older than other UKB ethnic groups (Table I). SC participants were shorter and lighter compared with White, Asian, and Black UKB participants (Table I). In contrast, SC and UKB Chinese participants shared similar height and weight in males and females.

### Prevalence of rHOA

JSN was less common in SC compared with White, Asian, and Black UKB participants (Table II). For all grades and locations, osteophytes were much rarer and smaller in SC, particularly in females (Supplementary Table 1). Likewise, the prevalence of rHOA, based on a combination of osteophyte grade and height-adjusted mJSW, was considerably lower in SC compared to White, Asian and Black UKB participants. Only 2.3% (95% Confidence interval [CI] 1.8%–2.8%) of SC females had evidence of rHOA, compared with 13.1% (12.6%–13.6%) of Whites in UKB, 14.0% (9.2%–20.2%) of Asians, 14.2% (8.8%–21.3%) of Black, and 1.5% (0.04%–8.3%) of Chinese (Table II). Though rHOA was more frequent in males, similar ethnic differences were seen, with rHOA in 12.0% (10.7%–13.3%) of SC males compared with 25.1% (24.5%–25.7%) of White UKB participants, 22.6% (17.7%–28.1%) of Asian, 24.4% (17.0%–33.1%) of Black, and 11.8% (4.4%–23.9%) of Chinese. Though the prevalence of rHOA in SC and Chinese UKB participants was similar, there were relatively few Chinese participants in UKB.

### Frequency of cam/pincer morphology and acetabular dysplasia

**Fig. 1:**
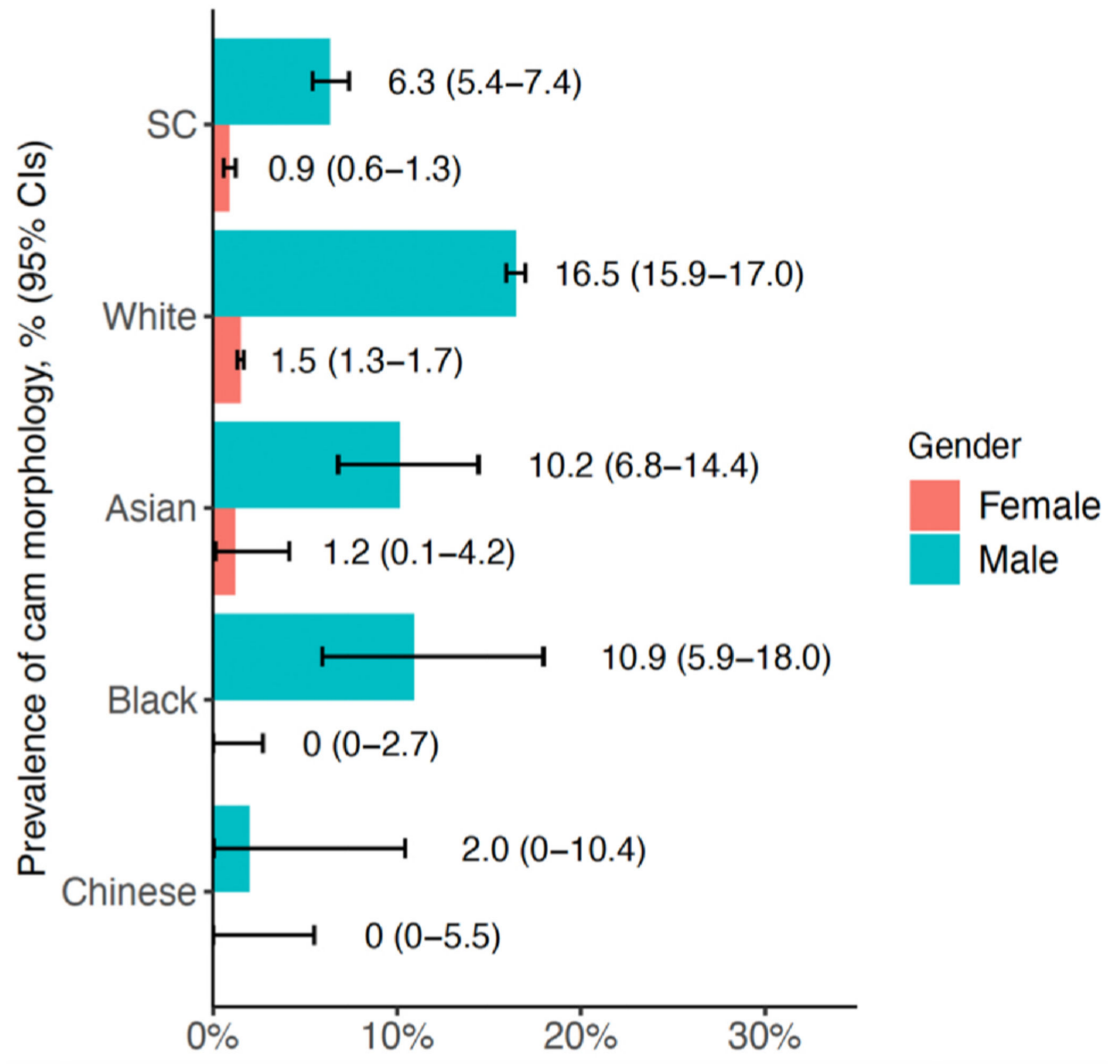
Proportion of cam morphology in SC and UKB participants. Prevalence of Cam morphology in SC cohort and UKB White/Asian/Black/Chinese participants, stratified by gender.

The mean value of AA in SC was lower than in White UKB participants, and similar to Chinese UKB participants (Table III). Cam morphology was considerably more common in males, with a low prevalence found in females of all ethnicities (Fig. 1). In males, cam morphology was less frequent in SC participants (6.3%, 95% CI 5.4%–7.4%) compared with UKB White (16.5%, 15.9%–17.0%), Asian (10.2%, 6.8%–14.4%) and Black (10.9%, 5.9%–18.0%) participants, but more frequent compared to UKB Chinese (2.0%, 0%–10.4%). Conversely, in females, acetabular dysplasia was more common in SC participants (7.6%, 6.7%–8.5%) compared to UKB White participants (6.1%, 5.7%–6.4%). The mean value of LCEA, and the prevalence of pincer morphology, showed less differences between groups. In females, SC (mean: 35.0 degrees, SD: 7.1 degrees) and UKB Chinese participants (mean: 32.2 degrees, SD: 6.7 degrees) had lower LCEA than UKB white participants (mean: 35.5 degrees, SD: 7.0 degrees), whereas no difference was seen in males.

### Hip shape

**Fig. 2:**
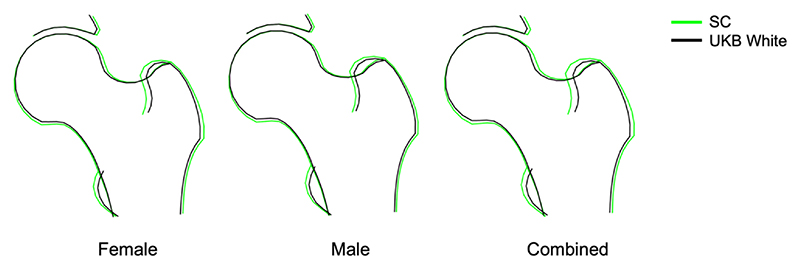
Comparison of mean hip shapes in SC and UKB White participants. Comparison of hip shape in SC and White UKB participants. Mean values from the first ten modes were used to draw composite hip shape, adjusted for differences in age, height, weight. Left: hip shape in females; middle: hip shape in males; right: hip shape in females and males combined. Green outline = SC cohort; black outline = UKB White participants.

The first 10 HSMs differed between SC and White UK participants in both sexes, with the greatest differences in mean HSM scores seen for HSM 1, 2, 3, and 6 (Supplementary Table 2). Similar differences were seen after results were centred on age, height and weight of White UKB participants (Supplementary Table 3). A composite hip shape was subsequently generated, combining the mean shapes of the first 10 normalised HSMs for each ethnic group, using White UK participants as the referent. In both sexes, SC participants had a wider femoral head width compared to White UKB participants (Fig. 2). SC participants also had larger lesser and greater trochanters, particularly in women. Similar differences in composite hip shape were observed, based on analysis of unadjusted HSMs (Supplementary Fig. 2). In terms of comparisons within UKB, HSM 2, 3 and 6 showed broadly similar differences between White and Chinese participants to those between White and SC participants, whereas differences in HSM1 were directionally opposite (Supplementary Tables 2 and 3). Several mode scores also differed between White, Asian and Black UKB participants. However, composite hip shape models showed little shape differences between ethnic groups.

### Hip geometry

Whereas hip shape from SSM is standardised for overall size, hip geometric measures were examined to evaluate differences in both size and shape, normalised for age, height and weight (Table IV) (unadjusted results are shown in Supplementary Table 4). FNW, HAL and DFH were smaller in females compared to males across all ethnicities. On the other hand, DFH/FNW ratio was greater in females. In both sexes, FNW and HAL were smaller in SC compared to White UKB participants, whereas DFH/FNW ratio was higher in SC. DFH was higher in SC compared to White UKB participants in females but similar in males. Asian, Black and Chinese UKB participants also had lower FNW and HAL compared to White participants, except for FNW in Chinese female and male participants. Compared to White UKB participants, DFH/FNW ratio was higher in male and female SC and Asians, higher in female Chinese and male Black participants, and similar in male Chinese and female Black participants.

## Discussion

This study presents a large multiethnic cross-sectional study, focusing on hip morphology and rHOA evaluation among 39,826 participants from UKB and 5924 participants from SC. We used high-resolution hip DXA scans to extract hip shape-related phenotypes and assess for rHOA, and standardised assessments were carried out in both cohorts to minimise bias in the evaluation criteria. We found that the prevalence of rHOA in SC is considerably lower than in those of European ancestry, reflecting a lower frequency of JSN and osteophytes, both of which were used to derive rHOA. This is consistent with an earlier report of a very low prevalence of rHOA among older Chinese subjects, compared with older US subjects.^1^

Several differences in hip morphology were observed between SC and White UKB participants which may have contributed to those in rHOA prevalence. For example, cam morphology, where the prominence of the superior aspect of the femoral neck has been suggested to lead to femoral acetabular impingement, was considerably more common in White UKB versus SC participants. Consistent with this observation, mean AA, an indicator of cam morphology, was higher in White participants. Several previous studies have reported cam morphology to be associated with a greater risk of HOA.^7,8,10,17^ On the other hand, cam morphology may be a feature of HOA in older adults, rather than having any causal role,^11^ and given the cross-sectional nature of our study it is possible some of the ethnic differences in hip morphology observed were a consequence rather than a cause of those in HOA prevalence. Though we found less difference in pincer morphology and acetabular dysplasia between ethnicities, previous reports suggest these features are less strongly associated with rHOA.^10^

In terms of other morphological differences, our finding that SC participants have a narrower femoral neck than White UKB participants may also contribute to differences in HOA prevalence, in light of previous evidence that greater FNW is associated with a higher risk of HOA.^12,23,24^ On visual inspection of composite SSM modes, no difference in FNW between SC and White UKB participants was evident, presumably due to correction for size differences by Procrustes analyses. Evaluation of DFH/FNW ratio suggested that SC participants have a greater DFH relative to FNW compared to White UKB participants. This difference may also have contributed to the lower HOA prevalence in SC, given our recent finding that lower DFH relative to FNW is associated with greater risk of rHOA, hospital-diagnosed HOA and total hip replacement in UKB.^13^ These observations are also consistent with previous findings from a case-control study that HOA cases had a lower femoral head-to-neck ratio compared to controls.^25^

There were only a small number of UKB participants from other ethnic groups. That said, a comparison of rHOA prevalence between White and Chinese UKB participants revealed similar differences to those seen in SC. In contrast, Asians had a similar rHOA prevalence to White UKB participants. Broadly similar differences were also observed when comparing hip geometry between White and Chinese UKB participants. As in SC, compared to White UK participants, Chinese UKB participants had a lower frequency of cam morphology (males only), narrower femoral neck and larger DFH/FNW ratio. Asian and Black males had a slightly lower prevalence of cam morphology compared to White participants, though this was higher than in Chinese. In Asian and Black UKB males, FNW was decreased relative to White participants, to a larger extent than Chinese participants. In female Asian and Black participants, DFH/FNW ratio showed a similar decrease relative to Chinese UKB participants, to that seen in White participants. However, an equivalent decrease was not seen in males, where DFH/FNW was increased relative to White participants to an equivalent extent to Chinese participants.

To some extent, SSM modes also showed similar differences between White and Chinese UKB participants to those between White UKB and SC participants. However, differences in HSM1 were directionally opposite, and little overall difference in shape was evident in composite shape models. This likely reflects the limited accuracy of shape estimates given the small number of Chinese UKB participants. Alternatively, as shown in Supplementary Fig. 1, a more negative value for HSM1, evident in SC scans, predominantly reflects larger lesser and greater trochanters. This could conceivably arise from systematic differences between cohorts in hip positioning during scan acquisition, given incomplete internal hip rotation increases the projection of the lesser and greater trochanters. That said, similar DXA scanning protocols were used in SC and UKB, making systematic differences in positioning unlikely. To the extent that lesser and greater trochanters are enlarged in SC participants, this could reflect greater strength of muscles attached to these sites, which could in turn lead to a reduced risk of HOA as a consequence of greater joint stability.

We are aware of two previous studies to have compared hip morphology between White and Chinese populations. Consistent with our findings, in a plain film radiographic study of 200 female participants, Dudda et al. reported a greater frequency of pathological impingement angle, reflecting asphericity of the femoral head equivalent to cam morphology, in older White compared with Chinese women without evidence of OA.^26^ Similarly, in a small Computed Tomography (CT)-based study of 201 younger subjects, AA, reflecting cam morphology, was found to be higher in White compared with Chinese subjects.^27^ In contrast, while these two studies suggested that pincer-type morphology, also thought to contribute to femoroacetabular impingement, is more common in White compared to Chinese subjects, we found no evidence of such a difference, based on LCEA. A reduced LCEA is also used to evaluate lateral acetabular coverage and dysplasia. Dudda et al^26^ and Van Houcke et al^27^ both found acetabular dysplasia to be more common in Chinese compared to White study participants, consistent with the present study where acetabular dysplasia was also more common in female SC versus White UKB participants.

Differences in HOA prevalence between Whites and Chinese could theoretically arise from environmental and/or genetic factors. The finding of similar ethnic differences within the UK points to genetic factors as the main explanation. In view of the important contribution of alterations in hip shape to HOA pathogenesis, and the ethnic differences in hip shape that we observed, it may be that such genetic differences act in part through altered hip shape. An indirect effect due to differences in height and weight, which are both known to contribute to HOA, is less likely given we adjusted for these parameters. Conversely, the relative lack of osteophyte formation in Chinese populations could be the primary genetic effect, with observed differences in hip shape a consequence rather than a cause. Consistent with this suggestion, our recent Mendelian Randomisation study suggested that cam morphology develops as part of or in parallel to the OA process, as opposed to playing a causal role.^11^ Likewise, our observed differences in FNW or DFH/FNW could conceivably reflect bone modelling and/or osteophyte changes consequent to HOA. Several other explanations for the ethnic differences in rHOA prevalence which we observed are also possible. These include greater weight and height of Caucasians, both of which are known risk factors for HOA.^28^ Though HOA may occur secondary to previous injury such as a hip fracture, previous hip fracture was an exclusion in both cohorts.

Our study has several strengths. First, we used two large, population-based cohorts, which provided more representative and reliable results compared to previous smaller studies. Second, we used advanced DXA-based machine-learning algorithms to assess hip morphology and rHOA. In terms of limitations, we recruited a community-based cohort in Shanghai but not a multi-regional collection that could reflect a broader picture of Chinese populations. In addition, any differences in study design may have contributed to the differences observed. SC is a single-centre study, with all participants invited for a DXA scan, while UK Biobank is a multi-centre study, with the expectation that 20% will be scanned in total. It is unclear how representative participants in the two cohorts who underwent DXA scans are, and to what extent any selection in the two cohorts is comparable.

Our study also had several other limitations. The small number of UKB Black, Asian, and Chinese participants meant that we had limited statistical power to compare non-Caucasian ethnic groups within UKB, resulting in substantial differences potentially going undetected. Though DXA-based measures of HOA prevalence and hip morphology were obtained using the same methods, using images obtained from identical scanners, the researchers responsible for annotation of images differed between SC and UKB. However, we attempted to minimise any systematic bias by having one of the annotators involved in the UKB DXA image analysis check the agreement with the SC annotators. Since analyses were based on the points available from annotation of the joint surface, hip geometric parameters, particularly HAL, could not be represented with complete accuracy. Furthermore, SSM has certain inherent limitations such as the model built depends on the images that are included, and the interpretation of single modes can be subjective, which is why we restricted interpretation of shape results to composite figures combining all modes. In addition, comparison of hip shape between SC and UKB could be confounded by systematic differences in hip positioning as only 2D images were available, particularly aspects such as trochanter size. Finally, our study only included cross-sectional data. Further, longitudinal studies are needed to assess the relationship between pre-existing differences in hip morphology and subsequent HOA development.

In conclusion, having measured rHOA and hip morphology on DXA images from the SC cohort, we compared results with those from UKB and found that rHOA prevalence is much lower in SC, due to a relative paucity of JSN and osteophytes. Several differences in hip morphology were identified, which may have contributed to this lower rHOA prevalence. These include lower prevalence of cam morphology, narrower femoral neck, and higher DFH/FNW ratio. To the extent that shape differences contribute to those in HOA prevalence, further investigation of these relationships, including longitudinal studies, is justified to help understand the role of changes in hip shape in the pathogenesis of HOA.

## Supplementary Material

**Appendix A. Supporting information**

Supplementary data associated with this article can be found in the online version at doi:10.1016/j.joca.2023.10.006.

Supplementary Material

## Figures and Tables

**Table I:** Descriptive statistics for UKB and SC cohorts.

	Female						Male				
	Shanghai Changfeng (n = 3417)	UK Biobank					Shanghai Changfeng (n = 2507)	UK Biobank			
		White (n = 20,374)	Asian (n = 171)	Black (n = 134)	Chinese (n = 65)			White (n = 18,646)	Asian (n = 266)	Black (n = 119)	Chinese (n = 51)
Age^a^ (years)	62.6 (9.4)	63.1 (7.4)	60.2 (7.9)	58.9 (7.0)	58.8 (7.3)		64.5 (9.7)	64.5 (7.6)	60.3 (8.5)	58.3 (7.3)	61.0 (6.5)
p-value^b^	0.004	–	**< 0.001**	**< 0.001**	**< 0.001**		1.000	–	**< 0.001**	**< 0.001**	0.004
p-value^c^	–	0.004	**0.001**	< 0.001	**0.001**		–	1.000	**< 0.001**	**< 0.001**	0.004
Height^a^ (cm)	156.8 (6.0)	163.7 (6.4)	158.4 (6.8)	163.9 (7.8)	158.9 (5.1)		168.1 (6.2)	177.3 (6.6)	172.2 (6.2)	176.6 (6.5)	169.9 (5.8)
p-value^b^	**< 0.001**	–	**< 0.001**	1.000	**< 0.001**		**< 0.001**	–	**< 0.001**	0.919	**< 0.001**
p-value^c^	–	**<** **0.001**	0.027	**< 0.001**	0.019		–	**< 0.001**	**< 0.001**	**< 0.001**	0.284
Weight^a^ (kg)	59.3 (9.1)	68.2 (12.8)	63.8 (11.0)	74.9 (15.0)	56.9 (8.2)		70.0 (9.9)	83.3 (13.4)	76.4 (12.1)	86.0 (14.2)	69.1 (9.9)
p-value^b^	**< 0.001**	–	**< 0.001**	**< 0.001**	**< 0.001**		**< 0.001**	–	**< 0.001**	0.345	**< 0.001**
p-value^c^	–	**< 0.001**	**< 0.001**	**< 0.001**	0.199		–	**< 0.001**	**< 0.001**	**< 0.001**	1.000

P values under adjusted P value threshold were shown in bold type.

a Mean (SD).

b One-way ANOVA, p-value when compared to the UKB White participants.

c One-way ANOVA, p-value when compared to the SC cohort. Bonferroni-corrected P value threshold was 0.00167 to adjust for multiple comparisons.

**Table II:** Prevalence (number (N) and (%)) of rHOA and JSN in UKB and SC cohorts.

		Female						Male				
		Shanghai Changfeng (n = 3417)	UK Biobank					Shanghai Changfeng (n = 2507)	UK Biobank			
			White (n = 20,374)	Asian (n = 171)	Black (n = 134)	Chinese (n = 65)			White (n = 18,646)	Asian (n = 266)	Black (n = 119)	Chinese (n = 51)
rHOA	**Minimal**	70 (2.0)	1768 (8.7)	17 (9.9)	7 (5.2)	1 (1.5)		267 (10.7)	2641 (14.2)	38 (14.3)	20 (16.8)	4 (7.8)
Category^a^	**Mild**	7 (0.2)	712 (3.5)	7 (4.1)	12 (9.0)	0 (0.0)		33 (1.3)	1528 (8.2)	20 (7.5)	9 (7.6)	2 (3.9)
	**Moderate**	1 (0.03)	146 (0.7)	0 (0.0)	0 (0.0)	0 (0.0)		0 (0.0)	393 (2.1)	2 (0.8)	0 (0.0)	0 (0.0)
	**Severe**	0 (0.0)	44 (0.2)	0 (0.0)	0 (0.0)	0 (0.0)		0 (0.0)	113 (0.6)	0 (0.0)	0 (0.0)	0 (0.0)
p-value^b^		**< 0.001**	–	0.811	0.025	0.091		**< 0.001**	–	0.466	0.519	0.426
p-value^c^		–	**< 0.001**	**< 0.001**	**< 0.001**	1.000		–	**< 0.001**	**< 0.001**	**< 0.001**	0.198
Total rHOA^a^		78 (2.3)	2670 (13.1)	24 (14.0)	19 (14.2)	1 (1.5)		300 (12.0)	4675 (25.1)	60 (22.6)	29 (24.4)	6 (11.8)
JSN Category^3^	**Mild**	39 (1.1)	1215 (6.0)	15 (8.8)	5 (3.6)	0 (0.0)		202 (8.1)	1976 (10.6)	33 (12.4)	14 (11.8)	4 (7.8)
	**Moderate to**	5 (0.1)	303 (1.5)	3 (1.8)	6 (4.5)	0 (0.0)		22 (0.9)	904 (4.9)	10 (3.8)	7 (5.9)	1 (2.0)
	**severe**											
p-value^b^		**< 0.001**	–	0.255	0.025	0.059		**< 0.001**	–	0.484	0.802	0.470
p-value^c^		–	**< 0.001**	**< 0.001**	**< 0.001**	1.000		–	**< 0.001**	**< 0.001**	**< 0.001**	0.489
Total JSN^a^		44 (1.3)	1518 (7.5)	18 (10.5)	11 (8.2)	0 (0.0)		224 (8.9)	2880 (15.5)	43 (16.2)	21 (17.7)	5 (9.8)

rHOA Category (grade 0–4): minimal = grade 1 only; mild = grade 2 only; moderate = grade 3 only; severe = grade 4 only; total rHOA = grade > 0. JSN Category (grade 0–3): mild = grade 1 only; moderate to severe = grade 2–3, total JSN = grade > 0. P values under adjusted P value threshold were shown in bold type.

a Number and %, N (%).

b Chi-squared test/Fisher’s exact test versus UK Biobank White participants on all outcome categories.

c Chi-squared test/Fisher’s exact test versus Shanghai Changfeng cohort on all outcome categories. Bonferroni-corrected P value threshold was 0.0025 to adjust for multiple comparisons.

**Table III:** Mean (SD) values and frequencies (N(%)) of cam/pincer morphology and acetabular dysplasia.

	Female						Male				
	Shanghai Changfeng (n = 3417)	UK Biobank					Shanghai Changfeng (n = 2507)	UK Biobank			
		White (n = 20,374)	Asian (n = 171)	Black (n = 134)	Chinese (n = 65)			White (n = 18,646)	Asian (n = 266)	Black (n = 119)	Chinese (n = 51)
AA (degrees)^a^	41.8 (4.7)	44.0 (5.9)	42.6 (3.6)	43.2 (3.7)	41.5 (3.3)		46.0 (8.1)	52.0 (13.2)	48.6 (11.0)	48.1 (10.6)	45.2 (6.7)
p-value^b^	**<** **0.001**	–	**< 0.001**	0.153	**< 0.001**		**< 0.001**	–	**< 0.001**	**< 0.001**	**< 0.001**
p-value^c^	–	**< 0.001**	0.999	0.090	0.299		–	**< 0.001**	**0.002**	0.325	0.990
LCEA (degrees)^a^	35.0 (7.1)	35.5 (7.0)	36.2 (6.8)	34.0 (6.9)	32.2 (6.7)		36.1 (6.8)	35.9 (7.0)	36.9 (7.2)	35.2 (7.0)	33.8 (6.7)
p-value^b^	**< 0.001**	–	1.000	0.101	**< 0.001**		1.000	–	0.211	1.000	0.351
p-value^c^	–	**< 0.001**	0.264	0.861	0.012		–	1.000	0.705	1.000	0.231
Cam (AA ≥ 60)^d^	30 (0.9)	302 (1.5)	2 (1.2)	0 (0.0)	0 (0.0)		159 (6.3)	3068 (16.5)	27 (10.2)	13 (10.9)	1 (2.0)
p-value^e^	**0.002**	–	0.535	0.136	0.379		**< 0.001**	–	**0.002**	0.062	**0.002**
p-value^f^	–	**0.002**	0.456	0.314	0.567		–	**< 0.001**	0.016	0.044	0.161
Pincer (LCEA≥45)^d^	267 (7.8)	1820 (8.9)	18 (10.5)	7 (5.2)	2 (3.1)		234 (9.3)	1802 (9.7)	35 (13.2)	14 (11.8)	2 (3.9)
p-value^e^	0.017	–	0.269	0.082	0.063		0.314	–	0.042	0.260	0.118
p-value^f^	–	0.017	0.130	0.175	0.111		–	0.314	0.033	0.228	0.136
Acetabular Dysplasia^d^	259 (7.6)	1235 (6.1)	8 (4.7)	12 (9.0)	9 (13.9)		146 (5.8)	1040 (5.6)	13 (4.9)	6 (5.0)	3 (5.9)
(LCEA < 25)											
p-value^e^	**< 0.001**	–	0.287	0.115	0.016		0.321	–	0.375	0.503	0.547
p-value^f^	–	**< 0.001**	0.098	0.324	0.059		–	0.321	0.323	0.459	0.579

Cam morphology was defined as AA ≥ 60 degrees. Pincer morphology was defined as LCEA ≥ 45 degrees. Acetabular dysplasia was defined as LCEA < 25 degrees. P values under adjusted P value threshold were shown in bold type.

a Mean (SD).

b One-way ANOVA, p value when compared to the UKB white participants.

c One-way ANOVA, p value when compared to the SC cohort.

d Number and %, N (%).

e Chi-squared test/Fisher’s exact test versus UK Biobank White participants.

f Chi-squared test/Fisher’s exact test versus Shanghai Changfeng cohort. Bonferroni-corrected P value threshold was 0.0025 to adjust for multiple comparisons.

**Table IV:** Hip geometry in UKB and SC cohorts.

	Female						Male				
	Shanghai Changfeng (n = 3417)	UK Biobank					Shanghai Changfeng (n = 2507)	UK Biobank			
		White (n = 20,374)	Asian (n=171)	Black (n = 134)	Chinese (n = 65)			White (n = 18,646)	Asian (n = 266)	Black (n=119)	Chinese (n = 51)
FNW (mm)^a^	28.8 (1.7)	29.0 (1.7)	28.0 (1.7)	28.2 (1.9)	28.3 (2.0)		33.7 (1.9)	34.6 (2.1)	33.1 (2.1)	33.0 (2.4)	33.8 (2.0)
p-value^b^	**<** **0.001**	–	**< 0.001**	**< 0.001**	0.003		**< 0.001**	–	**< 0.001**	**< 0.001**	0.067
p-value^c^	–	**< 0.001**	**< 0.001**	**< 0.001**	0.080		–	**< 0.001**	**< 0.001**	0.042	1.000
HAL (mm)^a^	88.2 (3.4)	90.9 (3.8)	88.9 (3.8)	88.1 (4.0)	88.0 (3.7)		99.3 (3.7)	103.2 (4.3)	100.4 (4.7)	100.8 (4.4)	100.0 (4.2)
p-value^b^	**< 0.001**	–	**< 0.001**	**< 0.001**	**< 0.001**		**< 0.001**	–	**< 0.001**	**< 0.001**	**< 0.001**
p-value^c^	–	**< 0.001**	0.184	1.000	1.000		–	**< 0.001**	0.004	0.005	0.946
DFH (mm)^a^	43.5 (1.8)	43.1 (1.8)	42.2 (2.0)	42.3 (2.0)	43.0 (1.9)		49.1 (1.9)	49.1 (2.1)	47.8 (2.1)	48.0 (2.4)	48.9 (2.1)
p-value^b^	**< 0.001**	–	**< 0.001**	**< 0.001**	1.000		0.341	–	**< 0.001**	**< 0.001**	1.000
p-value^c^	–	**< 0.001**	**< 0.001**	**< 0.001**	0.482		–	0.341	**< 0.001**	**< 0.001**	0.999
DFH/FNW ratio^a^	1.51 (0.07)	1.49 (0.07)	1.51 (0.07)	1.49 (0.08)	1.55 (0.08)		1.47 (0.07)	1.42 (0.07)	1.44 (0.07)	1.46 (0.08)	1.46 (0.08)
p-value^b^	**< 0.001**	–	**< 0.001**	0.987	**< 0.001**		**< 0.001**	–	**< 0.001**	**< 0.001**	0.007
p-value^c^	–	**< 0.001**	1.000	0.146	**0.001**		–	**< 0.001**	**< 0.001**	1.000	1.000

Adjusted mean and SD of hip geometry from different ethnic groups is shown, adjusted for age, height and weight. P values under adjusted P value threshold were shown in bold type.

a Mean (SD).

b One-way ANOVA, p value when compared to the UKB white participants.

c One-way ANOVA, p value when compared to the SC cohort. Bonferroni-corrected P value threshold was 0.00125 to adjust for multiple comparisons.

## Data Availability

The UK Biobank Study rHOA data from this study will be available in a forthcoming data release as it has been returned to the UK Biobank Study. Users must be registered with UK Biobank to access their resources (https://bbams.ndph.ox.ac.uk/ams/).
