# Supplementary material for: Comparison between UK Biobank and Shanghai Changfeng suggests distinct hip morphology may contribute to ethnic differences in the prevalence of hip osteoarthritis
Source: Osteoarthritis Cartilage. Author manuscript; Available in PMC 2026 Jan 6. (PMC7618574; doi:10.1016/j.joca.2023.10.006)
Supplement: Supplementary Material [file EMS211784-supplement-Supplementary_Material.docx]

Supplementary table 1 | Frequencies of osteophyte grade in UKB and SC cohorts

|  | | Female | | | | | Male | | | | |
| --- | --- | --- | --- | --- | --- | --- | --- | --- | --- | --- | --- |
|  | | Shanghai Changfeng  (n=3,417) | UK Biobank | | | | Shanghai Changfeng  (n=2,507) | UK Biobank | | | |
|  |  |  | White  (n=20,374) | Asian  (n=171) | Black  (n=134) | Chinese  (n=65) |  | White  (n=18,646) | Asian  (n=266) | Black  (n=119) | Chinese  (n=51) |
| Any OP location^1^ | **grade 1** | 30 (0.9) | 885 (4.3) | 6 (3.5) | 2 (1.5) | 1 (1.5) | 85 (3.4) | 1,419 (7.6) | 13 (4.9) | 9 (7.6) | 0 (0.0) |
|  | **grade 2** | 2 (0.06) | 450 (2.2) | 1 (0.6) | 5 (3.7) | 0 (0.0) | 2 (0.1) | 889 (4.8) | 8 (3.0) | 1 (0.8) | 1 (2.0) |
|  | **grade 3** | 0 (0.0) | 71 (0.3) | 1 (0.6) | 1 (0.7) | 0 (0.0) | 0 (0.0) | 208 (1.1) | 1 (0.4) | 0 (0.0) | 0 (0.0) |
| p-value^2^ | | **<0.001** | -- | 0.413 | 0.194 | 0.367 | **<0.001** | -- | 0.090 | 0.131 | 0.094 |
| p-value^3^ | | -- | **<0.001** | **<0.001** | **<0.001** | 1.000 | -- | **<0.001** | **<0.001** | 0.011 | 0.017 |
| Acet OP^1^ | **grade 1** | 23 (0.7) | 665 (3.3) | 6 (3.5) | 2 (1.5) | 1 (1.5) | 52 (2.1) | 838 (4.5) | 7 (2.6) | 5 (4.2) | 0 (0.0) |
|  | **grade 2** | 1 (0.03) | 330 (1.6) | 1 (0.6) | 2 (1.5) | 0 (0.0) | 2 (0.1) | 569 (3.1) | 6 (2.3) | 1 (0.8) | 1 (2.0) |
|  | **grade 3** | 0 (0.0) | 16 (0.1) | 1 (0.6) | 1 (0.7) | 0 (0.0) | 0 (0.0) | 102 (0.5) | 1 (0.4) | 0 (0.0) | 0 (0.0) |
| p-value^2^ | | **<0.001** | -- | 0.120 | 0.072 | 0.456 | **<0.001** | -- | 0.376 | 0.425 | 0.341 |
| p-value^3^ | | -- | **<0.001** | **<0.001** | **<0.001** | 0.377 | -- | **<0.001** | **<0.001** | 0.031 | 0.055 |
| Sup Fem OP^1^ | **grade 1** | 2 (0.06) | 495 (2.4) | 0 (0.0) | 1 (0.7) | 0 (0.0) | 14 (0.6) | 981 (5.3) | 5 (1.9) | 1 (0.8) | 0 (0.0) |
|  | **grade 2** | 0 (0.0) | 245 (1.2) | 0 (0.0) | 1 (0.7) | 0 (0.0) | 1 (0.04) | 457 (2.5) | 2 (0.8) | 0 (0.0) | 0 (0.0) |
|  | **grade 3** | 0 (0.0) | 48 (0.2) | 0 (0.0) | 0 (0.0) | 0 (0.0) | 0 (0.0) | 72 (0.4) | 0 (0.0) | 0 (0.0) | 0 (0.0) |
| p-value^2^ | | **<0.001** | -- | 0.091 | 0.475 | 0.358 | **<0.001** | -- | 0.019 | 0.043 | 0.208 |
| p-value^3^ | | -- | **<0.001** | 0.907 | 0.008 | 1.000 | -- | **<0.001** | 0.002 | 1.000 | 1.000 |
| Inf fem OP^1^ | **grade 1** | 6 (0.2) | 177 (0.9) | 0 (0.0) | 1 (0.7) | 0 (0.0) | 22 (0.9) | 567 (3.0) | 5 (1.9) | 3 (2.5) | 0 (0.0) |
|  | **grade 2** | 0 (0.0) | 52 (0.3) | 0 (0.0) | 2 (1.5) | 0 (0.0) | 1 (0.04) | 160 (0.9) | 1 (0.4) | 0 (0.0) | 0 (0.0) |
|  | **grade 3** | 0 (0.0) | 19 (0.1) | 0 (0.0) | 0 (0.0) | 0 (0.0) | 0 (0.0) | 69 (0.4) | 0 (0.0) | 0 (0.0) | 0 (0.0) |
| p-value^2^ | | **<0.001** | -- | 0.532 | 0.091 | 0.742 | **<0.001** | -- | 0.369 | 0.655 | 0.473 |
| p-value^3^ | | -- | **<0.001** | 0.746 | **<0.001** | 0.893 | -- | **<0.001** | 0.049 | 0.142 | 1.000 |

OP = osteophyte; Acet OP = acetabular osteophyte; Sup Fem OP = superior femoral head osteophyte; Inf fem OP = inferior femoral head osteophyte. ^1^Number and %, N (%); ^2^Chi-squared test / Fisher’s exact test versus UK Biobank White participants on all outcome categories; ^3^Chi-squared test / Fisher’s exact test versus Shanghai Changfeng cohort on all outcome categories. Bonferroni-corrected P value threshold was 0.00125 to adjust for multiple comparisons.

Supplementary table 2 | The first 10 hip shape mode scores in UKB and SC cohorts (unadjusted)

|  | Female | | | | | Male | | | | |
| --- | --- | --- | --- | --- | --- | --- | --- | --- | --- | --- |
|  | Shanghai Changfeng  (n=3,417) | UK Biobank | | | | Shanghai Changfeng  (n=2,507) | UK Biobank | | | |
|  |  | White  (n=20,374) | Asian  (n=171) | Black  (n=134) | Chinese  (n=65) |  | White  (n=18,646) | Asian  (n=266) | Black  (n=119) | Chinese  (n=51) |
| HSM 1^1^ | -0.36 (0.97) | 0.28 (0.92) | 0.39 (0.95) | 0.30 (0.96) | 0.54 (0.91) | -0.85 (0.95) | -0.31 (0.98) | -0.08 (1.00) | -0.22 (0.95) | -0.27 (1.10) |
| p-value^2^ | **3.5e-287** | -- | 0.715 | 1.000 | 0.223 | **1.4e-143** | -- | 0.001 | 1.000 | 1.000 |
| p-value^3^ | -- | **3.5e-287** | **1.6e-23** | **9.9e-15** | **2.2e-13** | -- | **1.4e-143** | **2.8e-33** | **9.9e-11** | **3.3e-04** |
| HSM 2^1^ | -0.50 (1.05) | 0.01 (0.99) | -0.60 (1.1) | -0.07 (0.97) | -0.20 (0.97) | -0.38 (1.01) | 0.02 (0.99) | -0.30 (1.08) | -0.01 (0.98) | -0.16 (0.89) |
| p-value^2^ | **1.4e-134** | -- | **8.7e-12** | 0.998 | 0.701 | **2.3e-80** | -- | **6.1e-07** | 1.000 | 1.000 |
| p-value^3^ | -- | **1.4e-134** | 0.973 | **5.8e-05** | 0.227 | -- | **2.3e-80** | 1.000 | 0.001 | 1.000 |
| HSM 3^1^ | 0.82 (0.96) | -0.30 (0.93) | -0.16 (0.92) | 0.38 (0.87) | 0.2 (1.09) | 1.14 (0.94) | 0.32 (0.96) | 0.36 (0.98) | 0.60 (1.08) | 0.86 (0.90) |
| p-value^2^ | **1.4e-267** | -- | 0.377 | **5.8e-16** | 0.002 | **1.1e-160** | -- | 1.000 | 0.013 | 0.001 |
| p-value^3^ | -- | **1.4e-267** | **1.5e-39** | **8.2e-07** | 0.001 | -- | **1.1e-160** | **2.1e-34** | **2.9e-08** | 0.388 |
| HSM 4^1^ | 0.00 (0.99) | -0.13 (0.99) | 0.18 (1.1) | -0.15 (1.00) | -0.39 (1.07) | 0.15 (0.92) | 0.14 (0.98) | 0.26 (0.98) | 0.36 (0.98) | -0.16 (0.86) |
| p-value^2^ | **5.7e-13** | -- | **4.3e-05** | 1.000 | 0.263 | 1.000 | -- | 0.359 | 0.155 | 0.162 |
| p-value^3^ | -- | **5.7e-13** | 0.063 | 0.494 | 0.009 | -- | 1.000 | 0.492 | 0.201 | 0.150 |
| HSM 5^1^ | -0.03 (0.93) | 0.04 (1.06) | 0.06 (1.05) | 0.0 (1.07) | -0.11 (0.95) | -0.45 (0.90) | -0.04 (0.93) | 0.09 (0.95) | -0.16 (0.96) | -0.42 (1.03) |
| p-value^2^ | 0.003 | -- | 0.997 | 0.988 | 0.899 | **2.0e-94** | -- | 0.196 | 1.000 | 0.032 |
| p-value^3^ | -- | 0.003 | 0.748 | 1.000 | 0.998 | -- | **2.0e-94** | **9.4e-19** | 0.008 | 1.000 |
| HSM 6^1^ | 0.60 (0.90) | 0.22 (0.98) | 0.60 (1.01) | 0.5 (1.06) | 0.78 (0.97) | 0.37 (0.89) | -0.26 (0.94) | 0.15 (0.96) | 0.23 (0.99) | 0.56 (1.03) |
| p-value^2^ | **3.2e-100** | -- | **4.9e-06** | 0.001 | **3.1e-05** | **2.2e-209** | -- | **2.8e-11** | **1.2e-07** | **5.0e-09** |
| p-value^3^ | -- | **3.2e-100** | 1.000 | 1.000 | 1.000 | -- | **2.2e-209** | 0.004 | 0.808 | 0.875 |
| HSM 7^1^ | 0.18 (0.91) | 0.08 (1.00) | 0.20 (1.06) | 0.42 (0.98) | 0.11 (1.00) | -0.23 (0.90) | -0.10 (0.99) | 0.12 (1.07) | 0.37 (1.19) | -0.29 (0.87) |
| p-value^2^ | **8.2e-07** | -- | 0.665 | 0.001 | 1.000 | **1.2e-09** | -- | 0.003 | **2.2e-06** | 1.000 |
| p-value^3^ | -- | **8.2e-07** | 1.000 | 0.051 | 1.000 | -- | **1.2e-09** | **2.0e-07** | **6.3e-10** | 1.000 |
| HSM 8^1^ | -0.20 (1.02) | -0.07 (0.98) | -0.09 (1.08) | -0.10 (1.2) | -0.19 (1.01) | -0.03 (1.01) | 0.08 (1.00) | -0.15 (1.09) | -0.03 (1.13) | 0.15 (1.23) |
| p-value^2^ | **9.9e-06** | -- | 1.000 | 0.998 | 0.967 | **3.5e-06** | -- | 0.002 | 1.000 | 1.000 |
| p-value^3^ | -- | **9.9e-06** | 1.000 | 1.000 | 1.000 | -- | **3.5e-06** | 0.660 | 1.000 | 1.000 |
| HSM 9^1^ | -0.13 (0.97) | -0.28 (0.93) | -0.29 (1.01) | -0.43 (0.93) | 0.03 (0.85) | 0.24 (0.98) | 0.31 (0.98) | 0.21 (0.96) | -0.05 (1.10) | 0.44 (0.88) |
| p-value^2^ | **4.6e-17** | -- | 0.996 | 0.444 | 0.046 | 0.006 | -- | 1.000 | 0.001 | 1.000 |
| p-value^3^ | -- | **4.6e-17** | 0.075 | 0.003 | 0.781 | -- | 0.006 | 1.000 | 0.019 | 1.000 |
| HSM 10^1^ | -0.07 (0.98) | 0.01 (0.95) | 0.01 (0.93) | -0.10 (0.95) | 0.10 (1.06) | 0.22 (1.01) | -0.01 (1.04) | -0.07(1.11) | -0.07 (1.14) | 0.42 (0.83) |
| p-value^2^ | **9.4e-06** | -- | 1.000 | 1.000 | 1.000 | **3.1e-25** | -- | 1.000 | 1.000 | 0.033 |
| p-value^3^ | -- | **9.4e-06** | 1.000 | 1.000 | 0.706 | -- | **3.1e-25** | **1.8e-04** | 0.033 | 1.000 |

Mean and SD of individual mode scores from different ethnic groups is shown. HSM = hip shape mode; SD = standard deviation. ^1^Mean (SD); ^2^One-way ANOVA, p value when compared to the UKB white participants; ^3^One-way ANOVA, p value when compared to the SC cohort. Bonferroni-corrected P value threshold was 0.0005 to adjust for multiple comparisons.

Supplementary table 3 | The first 10 hip shape mode scores in UKB and SC cohorts (adjusted)

|  | Female | | | | | Male | | | | |
| --- | --- | --- | --- | --- | --- | --- | --- | --- | --- | --- |
|  | Shanghai Changfeng  (n=3,417) | UK Biobank | | | | Shanghai Changfeng  (n=2,507) | UK Biobank | | | |
|  |  | White  (n=20,374) | Asian  (n=171) | Black  (n=134) | Chinese  (n=65) |  | White  (n=18,646) | Asian  (n=266) | Black  (n=119) | Chinese  (n=51) |
|  | Mean (SD) | | | | | Mean (SD) | | | | |
| HSM 1^1^ | -0.32 (0.94) | 0.28 (0.92) | 0.47 (0.94) | 0.31 (0.92) | 0.45 (0.88) | -0.86 (0.92) | -0.31 (0.98) | -0.13 (0.99) | 0.00 (0.91) | 0.04 (1.04) |
| p-value^2^ | **2.2e-264** | -- | 0.078 | 1.000 | 1.000 | **2.5e-155** | -- | 0.028 | 0.004 | 0.191 |
| p-value^3^ | -- | **2.2e-264** | **1.0e-26** | **7.1e-14** | **1.9e-10** | -- | **2.5e-155** | **3.2e-31** | **2.2e-20** | **4.2e-10** |
| HSM 2^1^ | -0.48(1.10) | 0.01 (0.99) | -0.55 (1.10) | -0.13 (0.94) | -0.27 (0.95) | -0.44 (1.01) | 0.02 (0.99) | -0.45 (1.06) | 0.04 (0.97) | -0.36 (0.88) |
| p-value^2^ | **1.8e-140** | -- | **3.6e-11** | 0.980 | 0.235 | **1.6e-107** | -- | **1.4e-13** | 1.000 | 0.051 |
| p-value^3^ | -- | **1.8e-140** | 0.696 | 0.0007 | 0.006 | -- | **1.6e-107** | 1.000 | **0.000** | 1.000 |
| HSM 3^1^ | 0.72 (0.95) | -0.30 (0.93) | -0.14 (0.90) | 0.36 (0.86) | 0.09 (1.09) | 1.02 (0.93) | 0.32 (0.96) | 0.28 (0.97) | 0.75 (1.07) | 0.35 (0.84) |
| p-value^2^ | **1.6e-259** | -- | 0.272 | **1.7e-15** | 0.007 | **4.5e-251** | -- | 1.000 | **8.1e-06** | 1.000 |
| p-value^3^ | -- | **1.6e-259** | **4.1e-31** | **1.6e-04** | **8.1e-07** | -- | **4.5e-251** | **2.9e-31** | 0.031 | **7.9e-06** |
| HSM 4^1^ | -0.16 (0.97) | -0.13 (0.99) | 0.33 (1.09) | -0.22 (0.98) | -0.54 (1.06) | 0.01 (0.91) | 0.14 (0.98) | 0.31 (0.98) | 0.42 (0.97) | -0.42 (0.85) |
| p-value^2^ | 1.000 | -- | **9.9e-09** | 1.000 | 0.263 | **2.1e-12** | -- | 0.048 | 0.022 | **3.5e-04** |
| p-value^3^ | -- | 1.000 | 0.063 | 0.494 | 0.009 | -- | **2.1e-12** | **2.4e-06** | **2.2e-05** | 0.012 |
| HSM 5^1^ | 0.08 (0.91) | 0.04 (1.06) | 0.10 (1.05) | -0.15 (1.07) | -0.11 (0.92) | -0.32 (0.90) | -0.04 (0.93) | 0.07 (0.95) | -0.14 (0.95) | -0.63 (1.02) |
| p-value^2^ | 0.003 | -- | 0.997 | 0.988 | 0.899 | **1.3e-45** | -- | 0.600 | 1.000 | **5.4e-05** |
| p-value^3^ | -- | 0.003 | 0.748 | 1.000 | 0.998 | -- | **1.3e-45** | **6.2e-10** | 0.296 | 0.188 |
| HSM 6^1^ | 0.54 (0.90) | 0.22 (0.98) | 0.58 (1.03) | 0.62 (1.10) | 0.67 (0.96) | 0.31 (0.88) | -0.26 (0.94) | 0.16 (0.95) | 0.15 (0.98) | 0.50 (1.03) |
| p-value^2^ | **5.7e-71** | -- | **1.4e-05** | **2.3e-05** | 0.002 | **1.1e-176** | -- | **4.4e-12** | **2.4e-05** | **8.9e-08** |
| p-value^3^ | -- | **5.7e-71** | 1.000 | 1.000 | 1.000 | -- | **1.1e-176** | 0.121 | 0.538 | 0.908 |
| HSM 7^1^ | 0.18 (0.90) | 0.08 (1.00) | 0.15(1.08) | 0.38 (0.97) | 0.04 (0.95) | -0.21 (0.89) | -0.10 (0.98) | 0.06 (1.07) | 0.29 (1.18) | -0.08 (0.84) |
| p-value^2^ | **4.4e-07** | -- | 0.665 | 0.001 | 1.000 | **1.6e-07** | -- | 0.197 | 0.004 | 0.759 |
| p-value^3^ | -- | **4.4e-07** | 1.000 | 0.051 | 1.000 | -- | **1.6e-07** | 0.001 | **2.6e-07** | 0.147 |
| HSM 8^1^ | -0.05 (1.01) | -0.07 (0.98) | 0.07 (1.04) | -0.25 (1.14) | -0.32 (1.04) | 0.12 (1.01) | 0.08 (1.00) | -0.16 (1.08) | -0.35 (1.06) | 0.87 (1.16) |
| p-value^2^ | 1.000 | -- | 1.000 | 1.000 | 0.967 | 0.975 | -- | 0.001 | **3.4e-05** | **1.9e-07** |
| p-value^3^ | -- | 1.000 | 1.000 | 1.000 | 1.000 | -- | 0.975 | **2.5e-04** | **8.3e-06** | **1.0e-06** |
| HSM 9^1^ | -0.10 (0.97) | -0.28 (0.93) | -0.46 (1.00) | -0.49 (0.92) | 0.15 (0.83) | 0.24 (0.96) | 0.31 (0.98) | 0.24 (0.95) | -0.03 (1.07) | 0.52 (0.87) |
| p-value^2^ | **2.2e-24** | -- | 0.996 | 0.444 | 0.046 | 0.025 | -- | 1.000 | 0.001 | 1.000 |
| p-value^3^ | -- | **2.2e-24** | 0.075 | 0.003 | 0.781 | -- | 0.025 | 1.000 | 0.024 | 0.433 |
| HSM 10^1^ | -0.05 (0.97) | 0.01 (0.95) | 0.05 (0.91) | -0.13 (0.94) | -0.01 (1.01) | 0.25 (1.01) | -0.01 (1.04) | -0.04 (1.10) | 0.19 (1.10) | 0.16 (0.77) |
| p-value^2^ | 0.004 | -- | 1.000 | 1.000 | 1.000 | **3.9e-33** | -- | 1.000 | 0.337 | 0.662 |
| p-value^3^ | -- | 0.004 | 1.000 | 1.000 | 0.706 | -- | **3.9e-33** | 0.0001 | 1.000 | 0.996 |

Mean and SD of residuals from different ethnic groups is shown. Mean values were adjusted for age, height and weight. HSM = hip shape mode; SD = standard deviation. ^1^Mean (SD); ^2^One-way ANOVA, p value when compared to the UKB white participants; ^3^One-way ANOVA, p value when compared to the SC cohort. Bonferroni-corrected P value threshold was 0.0005 to adjust for multiple comparisons.

Supplementary table 4 | Unadjusted hip geometry in UKB and SC cohorts

|  | Female | | | | | Male | | | | |
| --- | --- | --- | --- | --- | --- | --- | --- | --- | --- | --- |
|  | Shanghai Changfeng  (n=3,417) | UK Biobank | | | | Shanghai Changfeng  (n=2,507) | UK Biobank | | | |
|  |  | White  (n=20,374) | Asian  (n=171) | Black  (n=134) | Chinese  (n=65) |  | White  (n=18,646) | Asian  (n=266) | Black  (n=119) | Chinese  (n=51) |
| FNW (mm)^1^ | 27.5 (2.0) | 29.0 (2.0) | 27.1 (2.0) | 28.4 (2.3) | 27.0 (2.0) | 31.9 (2.2) | 34.6 (2.4) | 32.1 (2.4) | 32.6 (2.6) | 32.0 (2.1) |
| p-value^2^ | **<0.001** | -- | **<0.001** | **<0.001** | **<0.001** | **<0.001** | -- | **<0.001** | **<0.001** | **<0.001** |
| p-value^3^ | -- | **<0.001** | 0.105 | **<0.001** | 0.530 | -- | **<0.001** | 1.000 | 0.007 | 1.000 |
| HAL (mm)^1^ | 84.8 (4.2) | 90.9 (4.8) | 86.0 (5.1) | 88.3 (5.1) | 84.8 (4.4) | 94.9 (4.6) | 103.2 (5.4) | 97.5 (5.7) | 99.7 (5.1) | 95.5 (4.9) |
| p-value^2^ | **<0.001** | -- | **<0.001** | **<0.001** | **<0.001** | **<0.001** | -- | **<0.001** | **<0.001** | **<0.001** |
| p-value^3^ | -- | **<0.001** | 0.032 | **<0.001** | 1.000 | -- | **<0.001** | **<0.001** | **<0.001** | 1.000 |
| DFH (mm)^1^ | 41.8 (2.2) | 43.1 (2.2) | 40.9 (2.4) | 42.2 (2.5) | 41.6 (2.3) | 46.9 (2.4) | 49.1 (2.6) | 46.5 (2.5) | 47.5 (2.7) | 46.9 (2.1) |
| p-value^2^ | **<0.001** | -- | **<0.001** | **<0.001** | **<0.001** | **<0.001** | -- | **<0.001** | **<0.001** | **<0.001** |
| p-value^3^ | -- | **<0.001** | **<0.001** | 0.633 | 1.000 | -- | **<0.001** | 0.111 | 0.087 | 1.000 |
| DFH/FNW ratio^1^ | 1.52 (0.08) | 1.49 (0.07) | 1.51 (0.07) | 1.49 (0.08) | 1.54 (0.09) | 1.47 (0.07) | 1.42 (0.07) | 1.45 (0.08) | 1.46 (0.08) | 1.47 (0.08) |
| p-value^2^ | **<0.001** | -- | **<0.001** | 1.000 | **<0.001** | **<0.001** | -- | **<0.001** | **<0.001** | **<0.001** |
| p-value^3^ | -- | **<0.001** | 0.374 | **<0.001** | 0.371 | -- | **<0.001** | **<0.001** | 0.403 | 1.000 |

Mean and SD of hip geometry from different ethnic groups is shown. FNW = Femoral Neck Width; HAL = hip axis length; DFH = diameter of femoral head. ^1^Mean (SD); ^2^One-way ANOVA, p value when compared to the UKB white participants; ^3^One-way ANOVA, p value when compared to the SC cohort. Bonferroni-corrected P value threshold was 0.00125 to adjust for multiple comparisons.

Supplementary Figure 1 |HSM1-10 of SC generated from UKB hip shape model


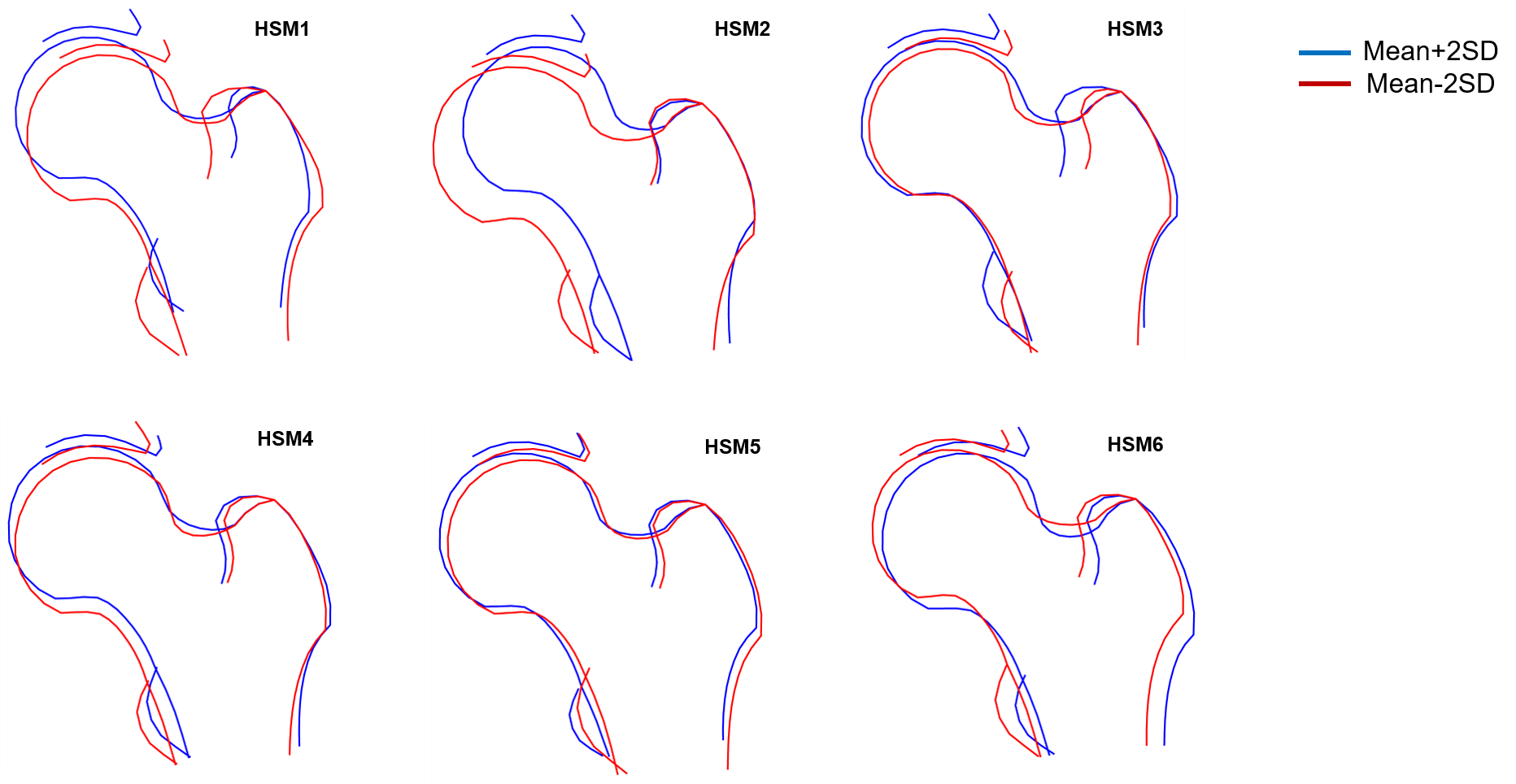


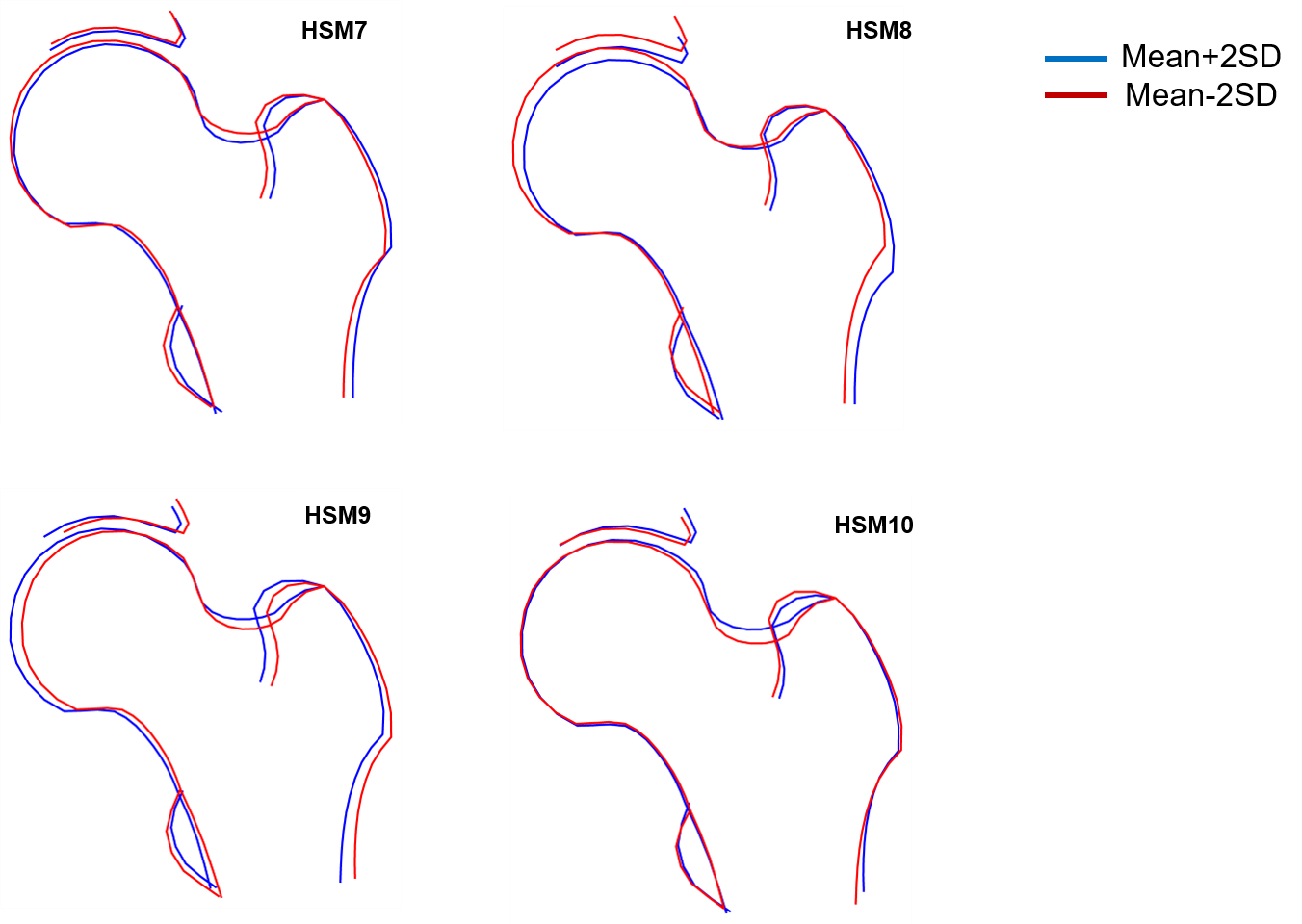


Supplementary Figure 2 | Unadjusted comparison of mean hip shapes in SC and UKB White participants


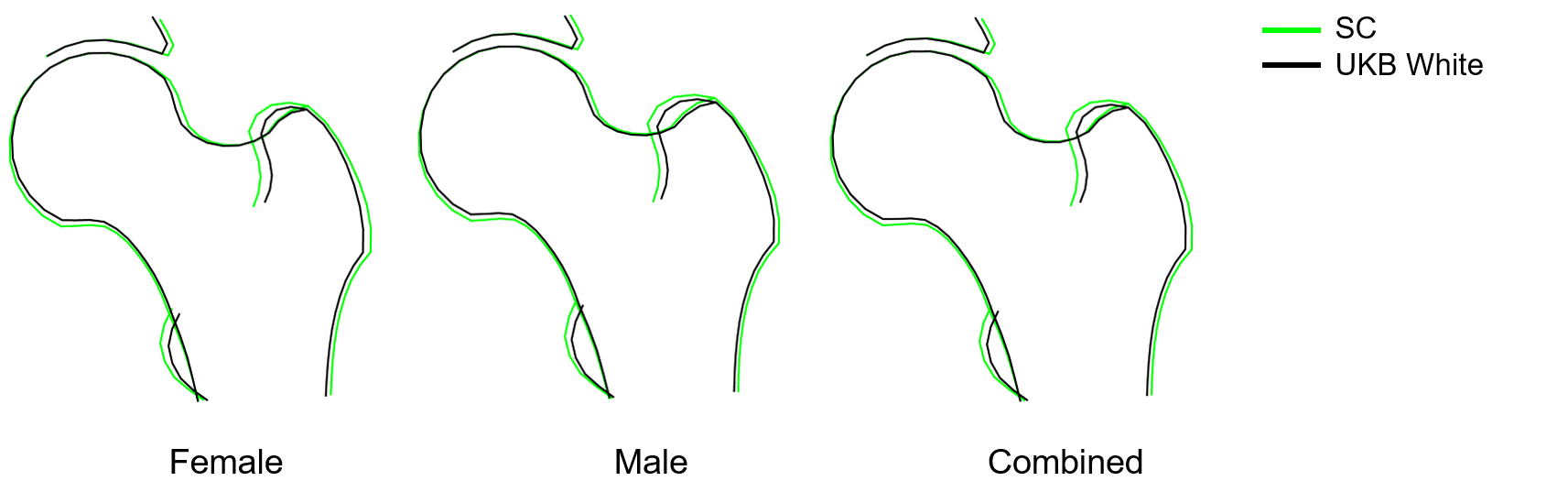


Supplementary Figure 3 | hip geometric parameter measurements (Heppenstall et al, 2023) Figure 2 DOI: 10.1101/2023.03.28.23287740;


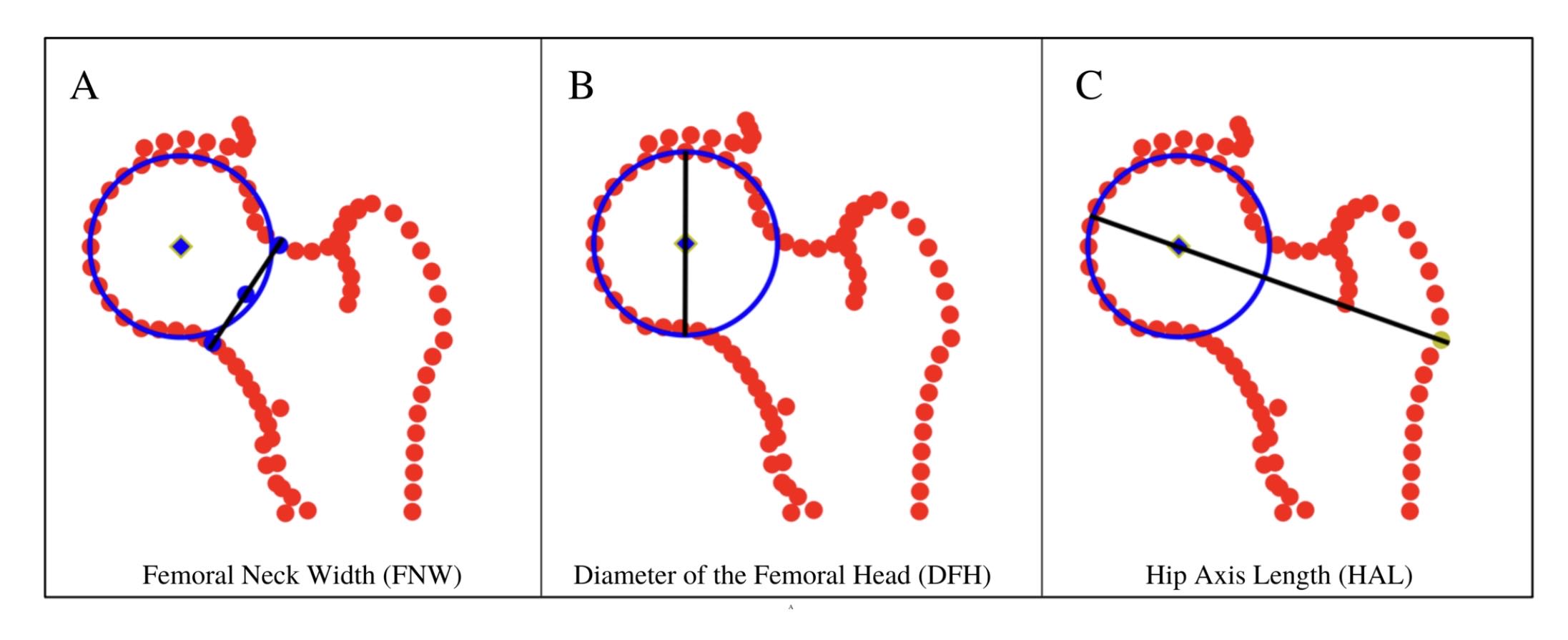


Geometric parameter measurements: A – Femoral Neck Width (FNW) derived using the line-segment method using points 6-12 on the medial side and points 32-38 on the lateral side, B – Diameter of the Femoral Head (DFH) derived by fitting a circle to points 15-28, C – Hip Axis Length (HAL) derived by finding the distance between points 49 and where the line intersects the circumference of the circle having passed through the centre of the femoral head.
